# Utility of fibrinolysis enhanced viscoelastic assays to evaluate fibrinolysis disorders in critically ill adults with severe infection: a scoping review

**DOI:** 10.1186/s13613-025-01528-x

**Published:** 2025-07-31

**Authors:** Matthew Self, Lucy A. Coupland, Anders Aneman

**Affiliations:** 1 Intensive Care Unit, Liverpool Hospital, Liverpool, Australia; 2 Ingham Institute for Applied Medical Research, Liverpool, Australia; 3 UNSW Medicine, University of New South Wales, Sydney, Australia

**Keywords:** Critical illness, Sepsis, Blood coagulation disorders, Fibrinolysis, Point-of-care technology, Thromboelastography

## Abstract

**Background:**

Acutely infected critically ill patients develop coagulopathies and perturbations to the fibrinolysis system that manifest as immunothrombosis. Whole blood viscoelastic testing, using an exogenous fibrinolytic agent to enhance fibrinolysis (FE-VET) can assess both processes of coagulation and fibrinolysis at the bedside. This scoping review aimed to illustrate clinical applicability, knowledge gaps and unmet needs for this emerging technology.

**Methods:**

A systematic search of bibliographic databases and the grey literature was performed between the 10th October 2024 and the 14th January 2025 using a pre-published protocol and reported according to the Preferred Reporting Items for Systematic Reviews and Meta-Analysis guideline for scoping reviews (PRISMA-ScR). Studies reporting FE-VET to investigate fibrinolysis in acutely infected patients admitted to the intensive care unit were assessed, including associations with disease severity and clinical outcomes.

**Results:**

The search identified 297 studies with 24 included in this review. Fifteen studies were observational (12 prospective, 3 retrospective), 4 case reports and series, 2 validation studies, 2 letters, and 1 poster abstract. No randomised controlled trials were identified. Most studies used varying concentrations of tissue plasminogen activator (tPA) to enhance fibrinolysis, with FE-VET performed at a single time point and the lysis time to achieve 50% reduction of maximum clot firmness being the most frequently reported variable. Fibrinolysis resistance was the prevailing state reported in acute sepsis or COVID-19 infections and associated with increased disease severity and worse clinical outcomes.

**Conclusion:**

Viscoelastic testing using a fibrinolysis enhancing agent demonstrated a spectrum of fibrinolysis resistance in acutely infected critically ill patients, associated with increased disease severity and mortality. Standardisation of the concentrations of fibrinolysis enhancing agents and the reporting of clot lysis parameters across testing devices are needed to establish reference values. This would improve future clinical studies of fibrinolysis, including trials of fibrinolytic therapies using a personalised medicine approach.

**Supplementary Information:**

The online version contains supplementary material available at 10.1186/s13613-025-01528-x.

## Introduction

Coagulopathies are prevalent in critically ill patients with acute infection and are associated with increased mortality [1–3]. Severe infections trigger a dysregulated host response involving a complex interplay between the immunological and coagulation systems [4, 5]. The resulting pathological state, known as immunothrombosis [4–6], is dynamic varying over time and with disease severity, and may be associated with a spectrum of haemostatic phenotypes ranging from hyper- to hypocoagulation and hyper- to hypofibrinolysis, depending on the underlying pathophysiology [7–11]. The proposed terminology for an acute state of reduced fibrinolytic activity is fibrinolysis resistance [12] hence, this is the term used in this review.

Historically, fibrinolysis testing, in particular assessing fibrinolysis resistance has trailed behind coagulation testing [13, 14]. This stems from the time-consuming methods required to study endogenous fibrinolysis (2–6 h in health) [13, 15]. Currently, there is no gold standard method to measure fibrinolysis [16], although several laboratory-based methods have been developed over time, each with their own benefits and limitations [17]. The fibrin degradation product, D-dimer, lacks specificity because it reflects both fibrin formation and breakdown [14, 18], and lacks the sensitivity to track dynamic changes in fibrinolysis because its half-life depends on renal clearance which may be compromised in critical illness [19]. Measuring plasma-based biomarkers such as plasminogen-activator inhibitor type 1 (PAI-1) to infer a mechanistic outcome risks ignoring the influence of all fibrinolysis regulators, fibrin clot structure and blood components on fibrinolysis [14, 16].

Point of care viscoelastic testing (VET), such as rotational thromboelastometry or thromboelastography (ClotPro^®^, ROTEM^®^, TEG^®^), overcomes several shortcomings of the plasma-based fibrinolysis measurements in the laboratory including the Euglobulin lysis time [17, 20]. VET requires minimal sample manipulation, analyses freshly collected whole blood, thus includes the contribution of platelet released fibrinolysis modulators, and provides a comprehensive assessment of both coagulation and fibrinolysis [20]. Nevertheless, there is minimal absolute difference in fibrinolysis parameters between healthy and infected individuals when standard VET assays are used [19, 21]. This is due to the slow rate of fibrinolysis occurring in health and, potentially, being further decreased by fibrinolysis inhibitors in acute infection [21, 22]. Fibrinolytic activators (e.g. tissue plasminogen activator, tPA, or urokinase) may be added to standard VET to enhance fibrinolysis such that, in healthy participants, complete clot lysis occurs within a clinically relevant runtime of 60 min. This strategy accentuates the difference in fibrinolysis between healthy and infected individuals which may increase the sensitivity to diagnose and differentiate between hyperfibrinolysis, normal fibrinolysis, fibrinolysis resistance and fibrinolysis shutdown [21, 22]. Fibrinolysis enhanced VET assays (FE-VET) have permitted investigations into the mechanisms, clinical correlations and potential treatments of fibrinolysis perturbations [21, 23]. The FE-VET may allow clinicians at the bedside to rapidly diagnose haemostatic phenotypes, monitor changes in fibrinolysis over time, risk stratify and potentially guide therapeutic strategies [3, 20, 21]. However, FE-VET is relatively new and variety in the VET technologies, inconsistencies in methodologies including the concentration of the fibrinolysis activator and the lack of reference values, render a systematic review of the literature challenging. The absence of prior reviews makes it unclear what the diagnostic, prognostic and therapeutic utilities are of using FE-VET in critically ill patients with acute infection. We therefore conducted a scoping review to systematically map the relevant research and to identify knowledge gaps that may direct future research. This scoping review aimed to illustrate whether FE-VET can detect changes in fibrinolysis in critically ill patients with acute infection and any associations with outcomes that could ultimately inform trials of clinical interventions.

## Methods

A scoping review was chosen as it provides the appropriate methodology to answer the broad research question, and would best allow mapping the key concepts and identifying knowledge gaps in an emerging field where FE-VET methods vary [24]. This review follows the Preferred Reporting Items for Systematic reviews and Meta-Analyses extension for Scoping Reviews (PRISMA-ScR) [25]. The review protocol has previously been published [26].

### Study eligibility criteria

To guide the search strategy, the *population* was defined as adult patients admitted to the intensive care unit (ICU) with acute infection (including bacterial, viral or fungal pathogens), the *concept* as assessment of fibrinolysis using VET which includes a fibrinolytic agent (e.g. tPA, urokinase or other) and the *context* as admission to ICU for management of an acute infection with all periods of follow up to assess associations with outcomes. No publication year or language restrictions were used. Non-English studies were translated using ChatGPT [27]. Translations were reviewed and validated by the authors to ensure accuracy and contextual relevance. Studies performed in animals, paediatric populations, using plasma-based clot lysis assays or experimental interventions such as infusion of endotoxin were excluded.

### Search strategy

An information specialist peer reviewed the search strategy that is available (MEDLINE format) in the review protocol [26]. The full search strategy for other databases is reported in the Additional File. Searches were conducted on 10th October 2024 and last updated on the 14th January 2025 in the following databases: MEDLINE, EMBASE, SCOPUS, the Cochrane library, and Epistemonikos. The grey literature was searched using Bielefeld Academic Search Engine (BASE), clinical trial registries, Google Scholar, as well as by hand searching reference lists and using forward citation search where possible within the electronic databases. All search outputs results were uploaded to Covidence (Veritas Health Innovation, Melbourne, Australia) [28].

### Study selection

Two authors (MS, AA) independently screened the titles and abstracts on Covidence, using a standardised screening template which contained inclusion and exclusion criteria. The same authors then reviewed the full texts of potentially relevant studies. Any disagreements regarding study selection and data extraction were resolved through discussion or by adjudication using an additional independent reviewer (LC). All study designs with results were included to comprehensively map the existing research, but ongoing studies with no results reported were excluded.

### Data extraction and presentation

A data extraction form was developed and published in the review protocol [26], modified with information on fibrinolysis measurements and timing, biomarkers and ICU length of stay added during data extraction. Both screening authors filled in the data extraction form in duplicate with the following information if available from sources: author(s), year and country of publication, funding, study design, participants and controls (if any), gender, age, type of infection, timing and type of FE-VET, fibrinolytic agent and concentration used, fibrinolysis measurements reported (e.g. lysis time, lysis onset time, clot lysis index or maximum lysis index), disease severity score(s), organ support requirements, follow up period, outcomes including thromboembolic and bleeding episodes, length of stay and mortality.

Patterns, themes and gaps in the literature relating to the review aims are described in Table 1. The scoping review will briefly discuss the quality of evidence, but in line with PRISMA-ScR guidelines, no formal quality and bias assessments were made [25].

**Table 1:** Knowledge gaps and recommendations

Areas with knowledge gaps	Recommendation
Testing methodology	Standardise testing methodology on different devices to use the same fibrinolytic agent (tPA), concentration and assay runtime that is optimal for the clinical context. Compare devices with similar methodology All lysis parameters should be reported and correlated with clinical outcomes to identify the optimal parameter
Timing of testing	Clearly report timing and consistency of testing. Using early, sequential testing allows further correlation with clinical outcomes
Standard definitions of fibrinolysis phenotypes and reference values	Build up dataset of comparable populations using standardised methodology to determine reference values in health and for different phenotypes of fibrinolysis resistance
Correlation to disease severity and biomarkers	Adequately powered studies with sample sizes of patients with a spectrum of disease aetiologies and severities Serial samples of fibrinolytic proteins, inflammatory and endothelial injury markers and correlation with FE-VET over time may better characterise the dynamic pathophysiology
Therapeutic implications	Consider usage of FE-VET as an enrichment strategy and monitoring tool to personalise therapies which target dysregulated fibrinolysis

## Results

**Fig. 1:**
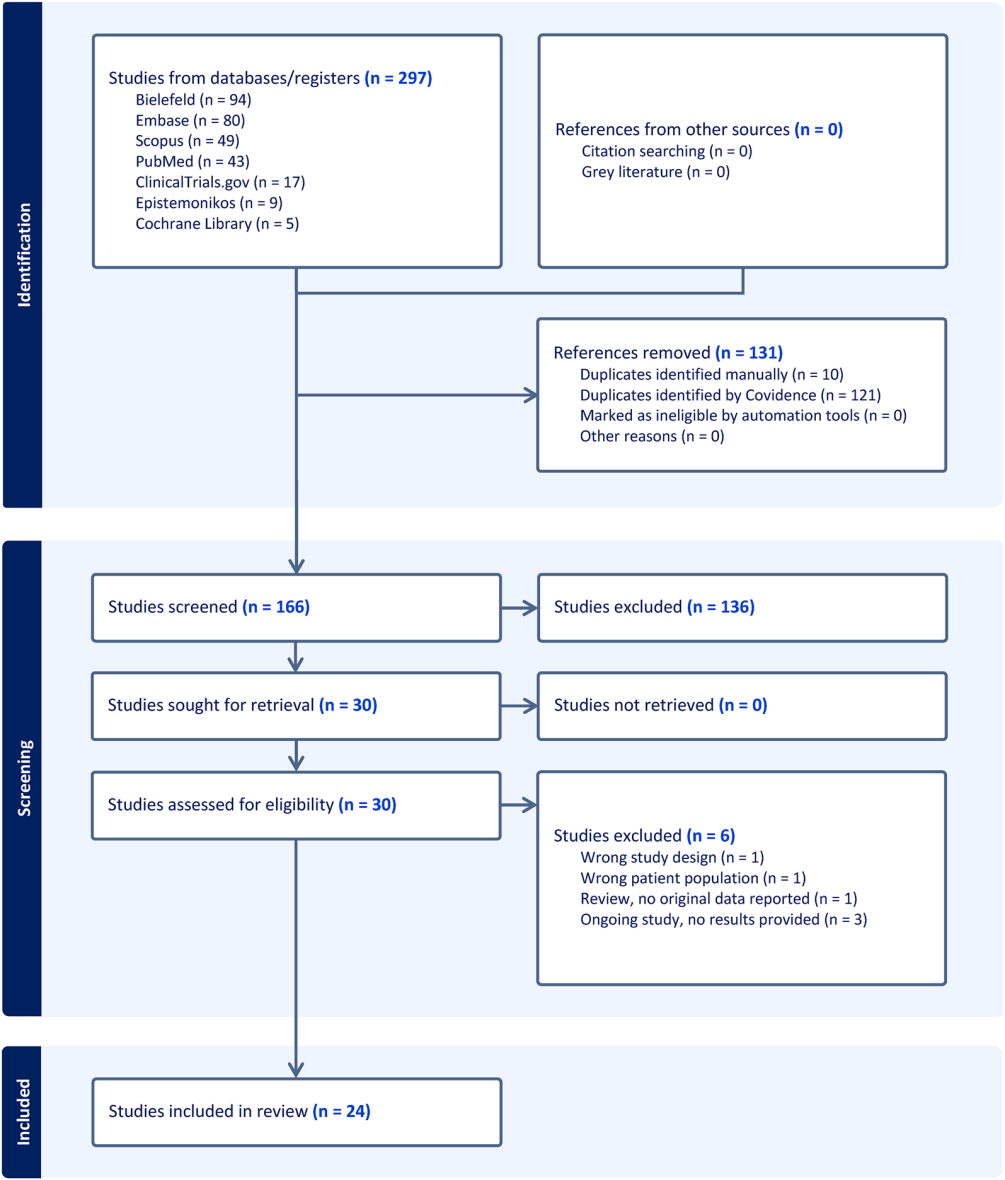
PRISMA Flowchart of study inclusion and exclusion

**Fig. 2:**
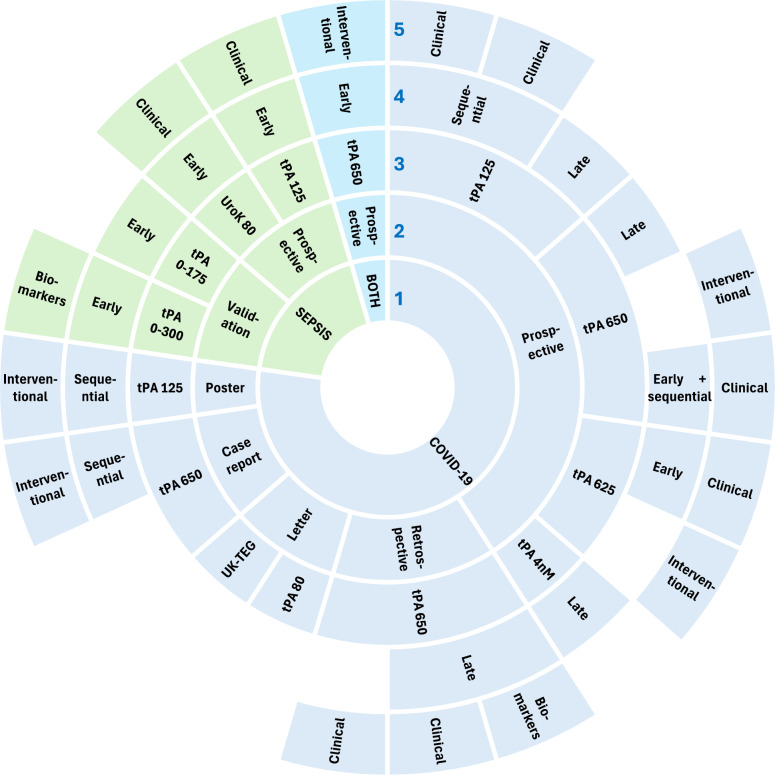
Summary of studies included in review. Doughnut pie chart depicting from innermost to outermost—Circle 1: ICU patient cohorts, including sepsis, COVID-19 or both conditions; Circle 2: Study design, including prospective, validation, case report, poster, letter; Circle 3: Fibrinolysis activator and concentration used, including tissue plasminogen activator (tPA; μg/mL) and Urokinase (Urok; U/mL); Circle 4: Frequency and timing of FE-VET relative to ICU admission, with early (0–3 days) and late (> 3 days); Circle 5: Study outcomes, including clinical, biomarkers or interventional

**Fig. 3:**
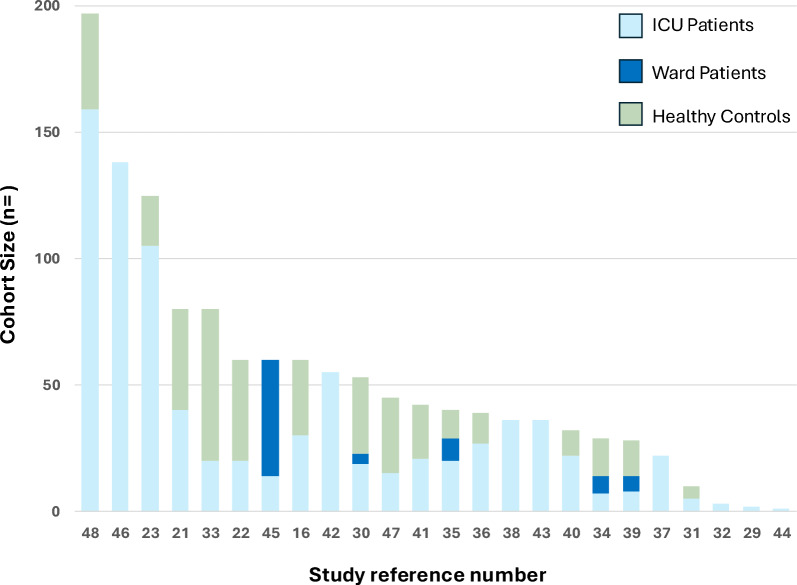
Cohort sizes in reviewed studies. Light blue bars indicate the number of ICU patients, Dark blue bars indicate Ward patients, and Green bars indicate the number of healthy controls

The PRISMA flowchart of the review process is shown in Fig. 1. A brief overview of the studies included in the review is provided in Table 2. The fibrinolysis parameters used in the studies and their definitions are provided in Table 3. The study designs, patient cohorts, fibrinolysis assay methodology and outcomes measured are summarised in Fig. 2. The number of patients and healthy controls included in each study are shown in Fig. 3. Additional File Table 1 provides greater detail on the above parameters as well as the VET results and key findings.

**Table 2:** Summary of studies included in scoping review—listed in order of year of publication

Author [REFs.]	Study design	FE-VET study cohort(s)	Device FE-VET agent	Assay timing median, frequency	Key findings
Panigada [21]	Prospective observational	Sepsis, n = 40 Healthy controls, n = 40	TEG^®^ Urokinase 80U/ml	< 24 h, once	• Fibrinolysis resistance predicted mortality and higher disease severity • FE-VET revealed a spectrum of fibrinolysis from near normal to severely impaired
Kuiper [22]	Technical, clinical validation	Sepsis, n = 20 Healthy controls, n = 40	ROTEM delta^®^ tPA 0, 125 or 175 ng/ml	< 24 h, once	• FE-VET separated fibrinolytic capacity of healthy controls and sepsis patients • Higher TPA concentration shortened LT
Bakchoul [29]	Case report	COVID-19 ICU, n = 2	ClotPro^®^ tPA 650 ng/ml	N/A	• Fibrinolysis resistance in COVID-19 ICU
Nougier [30]	Prospective observational case control	COVID-19 ICU, n = 19 COVID-19 ward, n = 4 Healthy controls, n = 10	ROTEM delta^®^ tPA 625 ng/ml	< 3 days, once	• Greater fibrinolytic impairment in COVID-19 cf. COVID ward and healthy controls • Greater impairment of fibrinolysis in patients with VTE
Weiss [31]	Prospective observational	COVID-19 ICU, n = 5 Healthy controls, n = 5	ROTEM delta^®^ tPA 80 ng/mL	N/A	• Fibrinolysis resistance in COVID-19 ICU
Zatroch [32]	Case series	COVID-19 ICU, n = 3	ClotPro^®^ tPA 650 ng/ml	Variable	• Sequential testing revealed prominent changes in fibrinolysis with LT changing the most compared to other VET parameters
Bachler [33]	Retrospective observational	COVID-19 ICU, n = 20 Healthy controls, n = 60	ClotPro^®^ TPA-test^®^ tPA 650 ng/ml	days, once	• Fibrinolysis resistance in COVID-19 ICU
Duque [34]	Case series	COVID-19 ICU, n = 7 COVID-19 ward, n = 7 Healthy controls, n = 15	ClotPro^®^ tPA 650 ng/ml	N/A, once	• Prolonged LT in COVID-19 vs healthy controls • No difference in LT between ICU and ward patients
Hammer [35]	Retrospective, observational	COVID-19 ICU, n = 20 COVID-19 Ward, n = 9 Healthy controls, n = 11	ClotPro^®^ tPA 650 ng/ml	N/A, once	• No significant differences observed in FE-VET parameters based on VTE or mortality
Heinz [36]	Prospective observational	COVID-19 ICU, n = 27 Healthy controls, n = 12	ClotPro^®^ tPA 650 ng/ml	7 days, once	• Fibrinolysis resistance in COVID-19 ICU
Hulshof [38]	Prospective longitudinal cohort	COVID-19 ICU, n = 36	ROTEM delta^®^ tPA 125 ng/ml	18 days, twice weekly	• Longitudinal analysis (6 wk) demonstrated persisting hypofibrinolysis despite anticoagulant treatment
Hulshof [37]	Prospective longitudinal cohort	COVID-19 ICU, n = 22	ROTEM delta^®^ tPA 125 ng/ml	25 days, twice	• Fibrinolysis resistance normalised 6 months after ICU discharge
Maier [39]	Prospective observational	COVID-19 ICU, n = 8 COVID-19 ward, n = 6 Healthy controls, n = 14	TEG 5000^®^ tPA 4 nM (~ 300 ng/mL)	8 days, once	• Fibrinolytic impairment only evident when tPA added to assay
Panigada [40]	Prospective observational	COVID-19 ICU, n = 22 Healthy controls, n = 10	TEG^®^ Urokinase 80U/ml	N/A, once	• Urokinase increased sensitivity to identify true hypofibrinolysis
Schrick [41]	Prospective observational	COVID-19 ICU, n = 21 Controls, n = 21	ClotPro^®^ tPA 650 ng/ml	N/A, once	• Decreased fibrinolytic response in aspirin non-responders cf. responders
Heubner [42]	Retrospective observational	COVID-19 ICU, n = 55	ClotPro^®^ tPA 650 ng/ml	17 days, once	• FE-VET did not differentiate survivors vs. non-survivors or predict bleeding complications • Impaired fibrinolysis in VTE patients
Nagy [43]	Poster abstract: Nested case control	COVID-19 ICU, n = 36	ROTEM delta^®^ tPA 125 ng/ml	3 weeks, weekly	• Shortest LT and lowest PAI-1 levels at week 1 in Dexamethasone + tocilizumab suggesting improved fibrinolysis
Coupland [23]	Prospective observational + exploratory interventional	COVID-19 ICU, n = 69 Non COVID-19 ICU, n = 36 Healthy controls, n = 20	ClotPro^®^ tPA 650 ng/ml	1 day, once	• Fibrinolysis resistance prevalent in COVID-19 and Non COVID-19 critically ill population • FE-VET can investigate cause of fibrinolytic impairment, guide therapy and monitor treatment effects
Forgács [44]	Case report	COVID-19 ICU, n = 1	ClotPro^®^ tPA 650 ng/ml	Daily, every 3 h	• Developed treatment algorithm using FE-VET to correct severe fibrinolysis using an alteplase infusion and fresh frozen plasma transfusions
Thaler [45]	Prospective observational	COVID-19 ICU, n = 14 COVID-19 ward, n = 46	ClotPro^®^ tPA 650 ng/ml	≤ 8 h and ≤ 24 h, twice	• Fibrinolysis resistance increased between hospital presentation and ICU admission • FE-VET did not predict who required ICU level care
Hulshof [46]	Prospective longitudinal cohort	COVID-19 ICU, n = 138	ROTEM delta^®^ tPA 125 ng/ml	15 days, weekly	• Serial VET showed dynamic changes, such that non-survivors displayed increasing fibrinolysis resistance
Rezigue [47]	Prospective experimental	COVID-19 ICU, n = 15 Healthy controls, n = 30	ROTEM delta^®^ tPA 625 ng/ml	N/A	• Hydroxyethyl starch (HES) enhanced tPA-induced fibrinolysis in patients with severe COVID-19 by reducing fibrin polymerisation
Brewer [48]	Prospective observational cohort	Sepsis, n = 30 Non-sepsis, n = 129 Healthy controls, n = 38	ROTEM delta^®^ tPA 125 ng/ml	< 24 h, once	• Fibrinolysis resistance occurred in sepsis and non-sepsis patients • Fibrinolysis resistance associated with increased mortality, disease severity and organ support
Scarlatescu [16]	Technical, clinical validation	Sepsis/septic shock, n = 30 Healthy controls, n = 30	ROTEM delta^®^ tPA 0, 100, 175, or 300 ng/mL	24-36 h, once	• Higher tPA concentrations (tPA 300 ng/ml) needed to determine the spectrum of fibrinolysis resistance, with complete clot lysis and shortened runtime

Original fibrinolysis terminology retained as per original reports. All assays referred to as fibrinolysis enhanced viscoelastometry (FE-VET). See Table 3 for definition of fibrinolysis parameters

*VTE* Venous ThromboEmbolism

^*^Septic shock 2005, **Surviving sepsis guideline 2012, ***Sepsis-3 definition

**Table 3:** Fibrinolysis variables reported in the reviewed studies and their definitions

Fibrinolysis parameter	Abbreviation	Definition
Clot lysis time	LT (ROTEM, ClotPro) CLT (TEG)	Time interval (sec or min) between the onset of clot formation and a 50%* reduction of the maximum clot firmness Time interval (sec or min) between the onset of clot formation and a 50% reduction of the maximum clot amplitude [41, 44] * some authors use 90% reduction of maximum clot firmness [40]
Lysis onset time	LOT	Time interval (sec or min) between the onset of clot formation and a 15% reduction of the maximum clot firmness [40]
Lysis index at 30 min	LI30% (ROTEM, ClotPro) Ly30% (TEG)	Reduction in clot amplitude 30 min after clotting onset relative to the maximum clot firmness [20] Reduction in clot amplitude 30 min after reaching MA [14, 41]
Maximum lysis	ML%	Reduction in clot amplitude relative to the maximum clot firmness at the completion of the assay [44]
Fibrinolysis speed		Speed of clot lysis (mm/min) between the LOT and the LT [25]
Kinetics of clot growth	t-AUCi	Time (sec) to maximal clot amplitude after reaching maximal clot formation velocity [20]

An initial 297 studies were identified with 24 included in the review, published between August 2015 [21] and April 2024 [16]. Twenty studies included COVID-19 patients [23, 29–47], 5 studies included sepsis patients [16, 21–23, 48] with one study including both aetiologies [23]. No studies investigating other viral or fungal infections were identified. Most studies originated in Europe, with one study from USA and one from Australia. All publications declared that there were no commercial or financial relationships that influenced their work.

### Study designs

Fifteen studies were observational (12 prospective, 3 retrospective) [21, 23, 30, 33, 35–39, 41, 42, 45–48], 4 case reports and series [29, 32, 34, 44], 2 validation studies [16, 22], 2 letters [31, 40], and 1 poster abstract [43]. No randomised controlled trials were identified.

Eight studies reported associations between fibrinolysis and clinical outcomes [21, 30, 35, 37, 42, 45, 46, 48]. Five studies evaluated the relationship between an intervention and fibrinolysis activity [23, 41, 43, 44, 47].

### Study participants and comparators

Most patients were male and over 60 years of age. Study cohorts were in general small with 20 studies including FE-VET results from less than or equal to 40 ICU patients and only 3 studies included more than 100 ICU participants [23, 46, 48]. Acutely infected patients were compared with non-age-matched healthy controls, ward patients, general ICU patients or internally depending on survivorship or fibrinolysis status. One study used age matched controls [41].

### Viscoelastic technology and fibrinolysis activator type and concentration

The most frequently reported FE-VET was the ClotPro^®^ TPA-test^®^ which contains 650 ng/mL tPA and was used in 11 studies [23, 29, 32–36, 41, 42, 44, 45]. Nine studies used the ROTEM delta^®^ with the EXTEM^®^ (extrinsic pathway) assay supplemented with varying concentrations of tPA: six studies used 125 ng/mL [22, 37, 38, 46, 48], one study used 80 ng/mL [31] and two studies used 625 ng/mL [30, 47]. Two studies used tPA titrations (0–300 ng/mL and 0–175 ng/mL, respectively) to study the impact of different tPA concentrations on fibrinolysis [16, 22]. Two studies used a modified kaolin activated TEG^®^ assay with 80 U/mL of urokinase (UK-TEG) in sepsis and COVID-19 ICU patients [21, 40]. One study used a modified kaolin activated TEG^®^ assay with 4 nM alteplase on platelet poor plasma [39].

### Timing of viscoelastic testing

Seven studies performed FE-VET within 3 days, commonly during the first 24 hr of ICU admission (early) [16, 21–23, 30, 45, 48] with 4 studies reporting FE- VET after 3 days of ICU admission (late) [33, 36, 39, 42]. Six studies performed sequential testing [32, 38, 43–46] and 1 study performed follow-up testing at 6 months [37]. Seven studies did not report specific timing of FE-VET analyses [29, 31, 34, 35, 40, 41, 47].

### Fibrinolysis assays, parameters and their definitions

The run times of the fibrinolysis assays varied across studies with a duration of [23, 42], [48], [16] and [22, 37, 38, 43, 46] min. The use of a heparin inhibitor, such as heparinase [21, 39, 40] or polybrene [23, 30, 36, 38, 41, 46], within the assay also varied between studies.

There was significant variation in the naming of the fibrinolysis parameters between technologies, the parameters reported by authors, as well as their definitions. Fibrinolysis parameters used in the reviewed literature are reported in Table 2.

Eleven studies defined fibrinolysis resistance to be present when the lysis parameter in patients was greater than the 90th to 97.5th percentile above the observed lysis parameter in healthy controls [16, 21–23, 30, 31, 33, 35, 40, 47, 48]. Eight studies did not provide a clear definition of fibrinolysis resistance [29, 34, 37–39, 43, 45, 46]. Fibrinolysis shutdown was most often defined as absent or incomplete clot lysis at 40 min which equated to a maximum clot firmness > 50% at this time point [23, 32, 42, 44].

### Fibrinolysis resistance incidence and disease severity scores

Prolonged fibrinolysis parameters were observed in ICU patients compared to healthy controls [16, 21–23, 30, 31, 33–36, 39, 41, 48]. Fibrinolysis resistance was reported for the majority of patients, irrespective of the timing of the assay relative to ICU admission, with an incidence as high as 80% (24/30) in sepsis [48] and 70% (14/20) in COVID-19 ICU patients [33]. A single study reported a 14% (3/22) incidence of fibrinolysis resistance in COVID-19 ICU patients [40]. Fibrinolysis resistance occurred in 55% (58/105) [23] and 61% (97/159) [48] of general ICU patients (including infectious and non-infectious). For non-infectious ICU patients, fibrinolysis resistance occurred in 57% (73/129) [48]. Higher disease severity (SOFA 6.5-9) was associated with increased incidence of fibrinolysis resistance [16, 21, 23, 33, 48] whilst lower disease severity (SOFA 3) was associated with a lower incidence of fibrinolysis resistance [40].

Two studies reported no significant difference in fibrinolytic function between ward and ICU patients with COVID-19 when timing was not specified [34, 35], similar results were found for one study when testing was conducted within 24 hr of hospital admission [45]. In contrast, one study performed testing within 3 days of admission and found significantly greater fibrinolysis resistance in COVID-19 ICU patients compared to COVID-19 ward patients [30].

Studies in which early and sequential testing was performed showed dynamic changes in fibrinolysis parameters. For example, testing between hospital admission (within 24 hr) and ICU admission (within 8 hr) revealed a rapid increase in fibrinolysis resistance [45]. Up to daily FE-VET in two case series reported that fibrinolysis became more resistant with increasing disease severity and improved with clinical recovery [32, 44]. One study, using twice weekly sampling a median of 18 days after intubation, showed that COVID-19 ICU patients had persistent fibrinolysis resistance over a 6-week period [38]. Longer term observational data indicated that fibrinolysis resistance normalised 6 months after ICU discharge when patients recovered from their acute infection [37].

Few studies identified hyperfibrinolysis, with two studies unable to detect hyperfibrinolysis [22, 35] and two studies reporting only sporadic cases, one with 2 non-sepsis patients [48], and the other with one COVID-19 ICU patient: LT 67 sec with the range in 20 healthy controls 132–227 sec [23].

### Fibrinolysis activity and disease biomarkers

Patients with fibrinolysis resistance had higher levels of C-reactive protein (CRP) [33, 48], leukocytes [48] plasmin-α2-antiplasmin complex [33], soluble thrombomodulin [33], PAI-1 [33, 35] and lower plasminogen levels [16], compared to patients with normal fibrinolysis. One study observed a significant positive correlation between PAI-1 activity and lysis time [35], whereas another study found no correlation [21]. One study found that patients with fibrinolysis resistance had significantly elevated platelet and fibrinogen levels [33], whilst other studies found no difference in platelet [21, 48] and fibrinogen levels [48]. There was no difference in D-dimer depending on fibrinolysis status [33, 48]. Two studies reported that patients with fibrinolysis resistance had significantly elevated lactate levels [21, 48] whilst one study found no difference [33].

### Fibrinolysis activity and clinical outcomes

Fibrinolysis resistance measured within 24 hr of admission was associated with higher disease severity (SOFA) and mortality for sepsis patients (7/18, 39%) [21] and in a combined population of sepsis and non-sepsis patients (30/97, 31%) [48]. Whereas one study found that FE-VET at intubation did not predict mortality in COVID-19 patients [46]. Instead, sequential testing at least once weekly, revealed that fibrinolysis resistance worsened over time in non-survivors (81/138, 59%) [46]. Studies which did not find a significant relationship between fibrinolysis resistance and mortality in COVID-19 patients performed late testing (median 17 days after symptom onset) (30/55, 55%) [42] or had a small sample size (5/20, 25%) [35].

One study reported that both septic and non-septic patients with fibrinolysis resistance had greater need for vasopressors, mechanical ventilation, renal replacement therapy and a longer ICU length of stay [48]. Conversely, another study in sepsis patients found no associations between fibrinolytic function and the incidence of acute kidney injury, duration of mechanical ventilation, or ICU length of stay [21].

Acutely infected ICU patients with fibrinolysis resistance were at greater risk of developing thromboses [30, 42, 48]. Conversely, one study found no significant difference between FE-VET results and thrombosis [35] and at 6 month follow-up, there was no difference in fibrinolytic function between ICU patients with or without thromboses diagnosed during admission [37]. The incidence of bleeding was reported to be higher in patients with fibrinolysis resistance [48], while another study found that VET performed later was unable to predict bleeding complications [42].

### Fibrinolysis activity and therapeutic interventions

One study demonstrated that fibrinolysis resistance could be corrected in 100% (32/32) of patients through supplementation of blood *ex-vivo* with additional tPA or plasminogen prior to FE-VET. The agent which was most effective in restoring fibrinolysis depended on the severity of fibrinolysis present, with the most severe requiring plasminogen [23]. Two case reports used FE-VET to monitor [23, 44] and titrate alteplase infusions [44]. Both studies demonstrated improvement in fibrinolysis resistance and one study reported marked improvement in oxygenation, lactate levels, liver function and resolution of portal vein thrombosis after normalising fibrinolysis [44]. A poster abstract highlighted that FE-VET could be used to monitor initial improvements in fibrinolysis resistance which subside over time following immunomodulatory therapy with tocilizumab and dexamethasone for COVID-19 ICU patients [43]. Aspirin non-responders displayed greater fibrinolysis resistance in COVID-19 ICU patients [41]. Infusion of the synthetic colloid hydroxyethyl starch (HES) enhanced fibrinolysis in COVID-19 ICU patients [47].

## Discussion

The findings of this scoping review were: (i) study design and assay methodology were variable; (ii) FE-VET detected changes in fibrinolysis activity in critically ill patients with acute infection; (iii) fibrinolysis resistance was common and associated with higher morbidity and mortality; (iv) there were limited interventional data regarding management of fibrinolysis resistance.

There was methodological heterogeneity between the retrieved studies because there have been no gold standard or reference values to define different fibrinolysis states in acute infection [23, 48]. In the body of research reviewed, concurrent or historic control groups were used to dichotomise patients with or without fibrinolysis resistance [16, 21–23, 30, 33, 35, 37, 38, 40, 41, 43, 44, 46, 48]. However, these definitions depend on the specific VET device used as well as the type and concentration of fibrinolysis enhancing agent. Additionally, the optimal FE-VET lysis parameter has not been established [48]. The most frequently reported parameter in this review was the lysis time which represented a 50% reduction in the maximum clot firmness after clotting onset. This review thus highlighted the lack of sensitivity and specificity data for FE-VET to diagnose fibrinolysis resistance based on the studies reported to date.

Higher concentrations of the fibrinolytic agent in FE-VET increased the propensity to identify fibrinolysis resistance, with the potential to separate patients into mild, moderate or severe fibrinolysis resistant phenotypes and shorten assay runtimes [16, 22, 23]. In the absence of tPA supplementation, standard VET did not reveal a pronounced difference in fibrinolysis between healthy controls and ICU patients with infection [16, 21, 22, 31, 39, 40]. Consequently, the methodology used to identify fibrinolysis resistance affected the reported incidence such that prior studies using standard VET identified 44% of COVID-19 patients [49] and 29% of sepsis patients [11] as having fibrinolysis resistance, whereas FE-VET identified 70% of COVID-19 [33] and 80% of sepsis patients [48] with fibrinolysis resistance. The sensitivity of FE-VET to identify the severity of fibrinolysis resistance was influenced by the dose of the fibrinolytic agent. Insufficiently enhanced fibrinolysis VET (tPA-125 ng/mL) resulted in the median lysis time approaching the assay runtime of 60–120 min in multiple studies [16, 37, 38, 43, 48] which meant that 56% of lysis times were unmeasurable in one study [38]. Consequently, insufficient fibrinolysis enhancement hides the full range of fibrinolysis resistance beyond the maximal runtime. In comparison, the higher concentration (tPA-650 ng/mL) uncovers a wide spectrum of fibrinolysis resistance, as fibrinolysis occurs more rapidly, leaving greater runtime to detect varying severities of fibrinolysis resistance which may reflect different pathophysiology, and hence treatment requirements, as well as patient outcomes. With tPA-650 ng/mL, the lysis time in healthy controls occurs within 300 sec [23], whereas in COVID and sepsis patients lysis times ranged between 67 and > 3600 sec [23, 33–36, 42, 45]. While this higher concentration of tPA enhances the detection of fibrinolysis resistance, there is a potential for reduced sensitivity to the severity of hyperfibrinolysis, however, this has not been demonstrated [22].

This review identified fibrinolysis resistance as the prevailing state associated with acute infection. Sepsis and COVID-19 infections share similar pathophysiology and commonly produce a hypercoagulable and fibrinolysis resistant state [6]. This state predisposes the increased formation and reduced breakdown of microvascular thrombi which could contribute to ischaemic multi-organ dysfunction [4, 5, 50], and may propagate the thrombo-inflammatory cascade [51] leading to overt disseminated intravascular coagulation (DIC) and death [3, 52–54]. The studies that included fibrinolysis biomarkers suggested that fibrinolysis resistance could be due to high thrombin generation [30] and hyperfibrinogenaemia [33] causing firm clots resistant to lysis, an excess of fibrinolysis inhibitors compared to activators [33, 35], and consumption or deficiency of fibrinolytic substrates, such as plasminogen [16, 19]. Hyperfibrinolysis was rarely observed, which is consistent with other literature [5, 8, 19]. This may be explained by the immuno-thrombotic state in acute infection shifting the balance towards fibrinolysis resistance [5, 14]. Hyperfibrinolysis might have been present very early in the disease trajectory, before FE-VET could be performed [23, 55], or late, during extreme manifestations such as DIC [5]. It is also possible that FE-VET loses the sensitivity to identify hyperfibrinolysis [22].

A third of the studies investigated associations between FE-VET and clinical outcomes. Overall, patients with fibrinolysis resistance tended to have increased mortality, extended length of stay, need for ICU organ support, disease severity, thromboses and bleeding. However, these associations varied between studies which might be explained by small sample sizes [35, 45], inconsistent definitions and measurement methodologies affecting the incidence of fibrinolysis resistance [21], and single samples taken very early [45] or late [42] providing less predictive power than sequential testing [46]. Larger study cohorts with higher incidences of fibrinolysis resistance and severe disease states are more likely to provide information on clinical associations [48]. Importantly, sequential FE-VET revealed the rapidly evolving changes in fibrinolysis over time, with worsening fibrinolysis resistance associated with increased disease severity [32, 44, 45] and mortality [42, 46].

Studies evaluating the impact of therapies on fibrinolysis resistance were infrequent, experimental and did not correlate findings with clinical outcomes. This included two small prospective observational studies [41, 47], two case reports [23, 44] and a poster abstract [43]. Given their low-quality and exploratory nature, any clinical benefit from therapies targeting fibrinolysis resistance remains to be determined.

Several recommendations for methodological development and future research may be made to reduce the gaps identified in this scoping review. The definitions of fibrinolysis parameters, the type and concentration of fibrinolysis enhancing agent and the runtimes should be standardised to utilise existing devices and technologies. All lysis parameters should be reported to identify those with the greatest correlation with clinical outcomes. Until the optimal fibrinolysis enhancing concentration is determined, we suggest absent or low (75–150 ng/mL) concentration of tPA to detect hyperfibrinolysis [22, 56, 57] and a higher (650 ng/mL) concentration of tPA for fibrinolysis resistance. A runtime of 60 min should be used because it provides information within a clinically relevant timeframe. A consistent body of data obtained with standardised methodology from comparable patient populations would inform definitions and reference values for different fibrinolysis states and levels of severity. A comparable plea was made to enable an increased understanding of fibrinolysis states in trauma [58], thus the establishment of methodological consistency across all disease states would be ideal. Similar to the studies reviewed, hyperfibrinolysis could be defined by a FE-VET lysis parameter that is below the 95% lower limit and fibrinolysis resistance occurring above the 95% upper limit for healthy controls. We suggest using the term severe fibrinolysis resistance to represent maximum lysis < 50% at the end of a 60 min runtime, instead of using fibrinolysis shutdown which has been specifically defined as reduced fibrinolysis after systemic activation of the fibrinolytic system [12]. Early fibrinolysis testing followed by sequential measurements, for example within 24 hr of admission and then daily while the patient requires active interventions to achieve a stable clinical condition, would enable the detection of dynamic fibrinolytic changes and prediction of clinical outcomes. Pairing FE-VET with fibrinolytic proteins (e.g. PAI-1, TAFI, α2AP), inflammatory biomarkers (e.g. CRP, procalcitonin, IL-2 and IL-6) and markers of endothelial injury (e.g. heparan sulfate, syndecan-1) would enable a description of coagulopathic phenotypes and their underlying pathophysiology. Profiling fibrinolysis phenotypes would define disease states and progression, and by extension inform therapeutic avenues.

The high incidence of fibrinolysis resistance and associations with worse outcomes in critically ill patients with acute infection, support the biological rationale and plausible benefit that interventions restoring fibrinolysis may assist many patients. Standardised reporting and usage of FE-VET would enable enrichment strategies for clinical trial populations or risk stratification. It would also allow selection of the most appropriate therapeutic intervention and dose for the patient to support a personalised medicine approach, with the response monitored in real-time at the point-of-care.

This scoping review has important strengths and limitations. Strengths include being based on a pre-published protocol and reporting using the PRISMA-ScR guidelines. Authors were contacted when there was missing study information and provided further data in some instances. The limitations of the scoping review relate to the heterogenous methodology between studies such as design, device, type and concentration of fibrinolytic agent, data definitions and timing. These methodological discrepancies limit the ability of the scoping review to draw strong conclusions from the data. No study compared different devices and only one study used the Euglobulin lysis time to compare results with FE-VET [22]. Most studies used small sample sizes with infrequent clinical events, thus risking both type I and type II statistical errors. The specific infectious aetiology was often sparsely reported, and it is unclear whether non-COVID-19 viruses or fungi affect fibrinolysis. Many studies performed single measurements at inhomogeneous stages of disease and few studies performed sequential tests which limits the assessment of dynamic changes in fibrinolysis. Most studies lacked age and sex-matched controls. Only two authors performed multivariate analyses on outcome data which resulted in a reduced effect estimate [46, 48]. This review is limited to acutely infected critically ill patients. Dysregulated fibrinolysis can also occur after trauma and elective surgery. Even though the underlying pathophysiology, sensitivity to tPA, degree of fibrinolysis resistance and clinical trajectory in these settings may differ from that seen during an acute infection, dysfunctional fibrinolysis is still associated with morbidity and mortality [5, 56, 59–61].

In conclusion, this scoping review found that using a fibrinolysis enhancing agent with viscoelastic testing (FE-VET) enabled the detection of a spectrum of fibrinolysis resistance in critically ill patients with acute infection. Fibrinolysis resistance was associated with increased disease severity and mortality. Future research should standardise the concentrations of fibrinolysis enhancing agents and the reporting of clot lysis parameters across testing devices to establish reference values for fibrinolysis resistance. Phenotyping fibrinolysis resistance in acutely infected critically ill patients could enrich clinical trial populations and enable an individualised approach to therapies which restore fibrinolysis.

## Supplementary Information

Supplementary material 1.

## Data Availability

The studies reported in this scoping review are available from bibliographic databases in the public domain.

## Abbreviations

α2AP: alpha-2 antiplasmin
APACHE: Acute physiology and chronic health evaluation
CRP: C-reactive protein
DIC: Disseminated intravascular coagulation
EX-A10: Clot amplitude 10 min after clotting time (CT) on Ex-test
FE-VET: Fibrinolysis enhanced viscoelastic testing
FIB-A10: Clot amplitude 10 min after clotting time (CT) on FIB-test
FR: Fibrinolysis resistance
hr: hour/s
ICU: Intensive Care Unit
IL: Interleukin
INR: International normalised ratio
IQR: Interquartile range
LT: Clot lysis time
min: minute/s
ng/mL: nanograms/millilitre
PAI-1: Plasminogen activator inhibitor-1
PRISMA-ScR: Preferred reporting items for systematic reviews and meta-analysis—scoping review
PT: Prothrombin time
ROTEM^®^: Rotational thromboelastometry
sec: second/s
SIC: Sepsis induced coagulopathy
SOFA: Sequential organ failure assessment
TAFI: Thrombin activatable fibrinolysis inhibitor
TEG^®^: Thromboelastography
tPA: Tissue plasminogen activator
TPA-LT: Clot lysis time obtained from TPA-test
U: International units
UK: Urokinase
VET: Viscoelastic testing
yr: years
