# Supplementary material for: Utility of fibrinolysis enhanced viscoelastic assays to evaluate fibrinolysis disorders in critically ill adults with severe infection: a scoping review
Source: Ann Intensive Care. 2025 Jul 31;15:110. doi: 10.1186/s13613-025-01528-x (PMC12314158; doi:10.1186/s13613-025-01528-x)
Supplement: Supplementary file 1 — Supplementary material 1. [file 13613_2025_1528_MOESM1_ESM.docx]

**Additional File:**

**Utility of fibrinolysis enhanced viscoelastic assays to evaluate fibrinolysis disorders in critically ill adults with severe infection: a scoping review.**

Matthew Self^1^, Lucy A. Coupland^1,2,3^, Anders Aneman^1,2,3^

^1^ Intensive Care Unit, Liverpool Hospital, Liverpool, Australia

^2^ Ingham Institute for Applied Medical Research. Liverpool, Australia

^3^ UNSW Medicine, University of New South Wales, Sydney, Australia

**Contents**

Database Search Strategy Pages 2-7

Table 1 – More detailed summary of included studies Pages 8-16

**Search Strategy for Scoping Review**

Initial search: 11^th^ October 2024

Last updated: 14^th^ January 2025

**MEDLINE**

*Population*

“critically ill” OR “critical illness” OR “intensive care*” OR “critical care*” OR “critically unwell” OR "Intensive Care Units"[Mesh] OR "Critical Care"[Mesh] OR "Critical Illness"[Mesh]

*Concept*

("Fibrin"[Mesh] OR "Fibrinogen"[Mesh] OR "Fibrinolysis"[Mesh] OR "Fibrin Clot Lysis Time"[Mesh] OR "Blood Coagulation Tests"[Mesh] OR "Blood Coagulation Factors"[Mesh] OR "Blood Coagulation"[Mesh] OR "Blood Coagulation Disorders"[Mesh]OR “fibrino*” OR “fibrinolytic resistance*” OR “fibrinolytic impairment*” OR “hypofibrinoly*” OR “hyperfibrinoly*” OR “fibrinolytic system*” OR “fibrinolytic activit*” OR “fibrinolysis shutdown” OR “fibrinolysis shut down” OR “fibrinolytic shutdown” OR “fibrinolytic shut down”)

AND

("Thrombelastography"[Mesh] OR "Whole Blood Coagulation Time"[Mesh] OR "Point-of-Care Systems"[Mesh] OR "Point-of-Care Testing"[Mesh] OR viscoelastic testing OR "viscoelastic test*" OR thromboelast* OR TEG OR "rotational thromboelast*" OR ROTEM OR “ClotPro” OR sonorheometry OR Quantra)

AND

("Urokinase-Type Plasminogen Activator"[Mesh] OR "Plasminogen Activators"[Mesh] OR "Tissue Plasminogen Activator"[Mesh] OR "Plasminogen"[Mesh] OR "Thrombolytic Therapy"[Mesh] OR "Fibrinolytic Agents"[Pharmacological Action] OR "Fibrinolytic Agents"[Mesh] OR “urokinase” OR “alteplase” OR “tPA” OR “tPA assay”)

*Context*

("Sepsis"[Mesh] OR "Shock, Septic"[Mesh] OR "Systemic Inflammatory Response Syndrome"[Mesh] OR "Bacteremia"[Mesh] OR "Infections"[Mesh]) OR ("COVID- 19"[Mesh] OR "SARS-CoV-2"[Mesh] OR "Coronavirus Infections"[Mesh] OR "Pneumonia, Viral"[Mesh] OR “coronavirus” OR “coronavirus disease 2019” OR “COVID- 19” OR “severe acute respiratory syndrome coronavirus 2”)

**EMBASE**

*Population*

intensive care/ or intensive care medicine/ or intensive care unit/ or medical intensive care unit/ or critical illness/ or critical care medicine/ or ("critical ill*" or "intensive care" or ICU or "critical care").mp.

*Concept*

fibrinolysis/ or blood clotting/ or blood clot lysis/ or fibrin clot/ or fibrinolysis/ or euglobulin lysis test/ or fibrin degradation product/ or ("fibrino*" or "fibrinolytic resistance*" or "fibrinolytic impairment*" or "hypofibrinoly*" or "hyperfibrinoly*" or "fibrinolytic system*" or "fibrinolytic activit*" or "fibrino* shutdown").mp.

AND

thromboelastography/ or thromboelastograph/ or elastography/ or thromboelastometry/ or "point of care system"/ or "point of care testing"/ or rapid on-site evaluation/ or ("Viscoelastic test*" or thromboelast* or TEG or "rotational thromboelast*" or ROTEM or ClotPro or sonorheometry or Quantra).mp.

AND

plasminogen/ or plasminogen activation/ or plasminogen activator/ or tissue plasminogen activator/ or fibrinolytic agent/ or alteplase/ or urokinase/ or anistreplase/ or tenecteplase/ or streptokinase/ or ("tpa" or "tpa assay").mp.

*Context*

infection/ or systemic inflammatory response syndrome/ or cytokine release syndrome/ or sepsis/ or septic shock/ or septicemia/ or severe sepsis/ or urosepsis/ or sepsis-associated coagulopathy/ or bacteremia/ or bloodstream infection/ or fungemia/ or candidemia/ or candidiasis/ or viremia/ or severe acute respiratory syndrome/ or virus pneumonia/ or coronavirus infection/ or coronavirus disease 2019/ or COVID-19 pneumonia/ or covid-19-associated coagulopathy/

**SCOPUS**

*Population*

TITLE-ABS-KEY ( intensive AND care OR "intensive care medicine" OR "intensive care unit*" OR icu OR "medical intensive care unit" OR "critical ill*" OR "critically ill" OR "critical care" OR "critical care medicine" )

*Concept*

TITLE-ABS-KEY ( fibrinoly* OR fibrino* OR hypofibrinoly* OR hyperfibrinoly* OR "blood clot lysis" OR "blood clotting" OR "fibrinolytic activit*" OR "fibrinolytic system*" OR "fibrinolytic shutdown" OR "fibrinolytic resistance" OR "fibrinolytic impairment" )

AND

TITLE-ABS-KEY ( thromboelasto* OR viscoelastic* OR rotem OR teg OR clotpro OR quantra OR sonorheometry )

AND

TITLE-ABS-KEY ( plasminogen OR "plasminogen activation" OR "plasminogen activator" OR "tissue plasminogen activator" OR "thrombolytic therapy" OR "fibrinolytic agent" OR alteplase OR urokinase OR anistreplase OR tenecteplase OR streptokinase OR tpa OR "tpa assay" )

*Context*

TITLE-ABS-KEY ( infection OR "systemic inflammatory response syndrome" OR "cytokine release syndrome" OR sepsis OR "septic shock" OR bacteremia OR "bloodstream infection" OR fungemia OR candidemia OR candidiasis OR viremia OR septicemia OR "severe sepsis" OR urosepsis OR "sepsis-associated coagulopathy" OR "severe acute respiratory syndrome" OR "virus pneumonia" OR coronavirus OR covid OR "coronavirus infection" OR "coronavirus disease 2019" OR "COVID-19 pneumonia" OR "covid-19-associated coagulopathy" )

**The Cochrane library**

*Population*

MESH: intensive care units, critical care, critical illness, multiple organ failure

KEYWORD: intensive care or "intensive care medicine" or "intensive care unit" or ICU or "medical intensive care unit" or "critical illness" or "critically ill" or "critical care" or "critical care medicine"

*Concept*

MESH: fibrinolysis, fibrin clot lysis time, blood coagulation, thromboelastography, plasminogen activators, urokinase-type plasminogen activator, plasminogen, tissue plasminogen activator, fibrinolytic agent, tenecteplase, streptokinase, thrombolytic therapy

KEYWORD: (fibrinoly* OR fibrino* OR hypofibrinoly* OR hyperfibrinoly* OR "blood clot lysis" OR "blood clotting" OR "fibrinolytic activity" OR "fibrinolytic system" OR "fibrinolytic shutdown" OR "fibrinolytic resistance" OR "fibrinolytic impairment") AND (thromboelasto* OR viscoelastic* OR ROTEM OR TEG OR ClotPro OR Quantra OR Sonorheometry) AND (plasminogen or "plasminogen activation" OR "plasminogen activator" OR "tissue plasminogen activator" OR "thrombolytic therapy" OR "fibrinolytic agent" OR alteplase OR urokinase OR anistreplase OR tenecteplase OR streptokinase OR tpa OR "tpa assay")

*Context*

MESH: infection/ or systemic inflammatory response syndrome/ or cytokine release syndrome/ or sepsis/ or septic shock/ or bacteremia/ or fungemia/ or candidemia/ or candidiasis/ or viremia/ or virus disease/ or severe acute respiratory syndrome/ or severe acute respiratory syndrome-related coronavirus/ or coronavirus/ or coronavirus infection/ or COVID-19/

KEYWORD: infection OR "systemic inflammatory response syndrome" OR "cytokine release syndrome" OR sepsis OR "septic shock" OR bacteremia OR "bloodstream infection" OR fungemia OR candidemia OR candidiasis OR viremia OR septicemia OR "severe sepsis" OR urosepsis OR "sepsis-associated coagulopathy" OR "septic complication" OR "severe acute respiratory syndrome" OR "virus pneumonia" OR coronavirus OR covid OR "coronavirus infection" OR "coronavirus disease 2019" OR "COVID-19 pneumonia" OR "covid-19-associated coagulopathy" OR "covid-19 associated coagulopathy"

**Epistemonikos**

*Population*

intensive care or "intensive care medicine" or "intensive care unit" or ICU or "medical intensive care unit" or "critical illness" or "critically ill" or "critical care" or "critical care medicine"

*Concept*

fibrinoly* OR fibrino* OR hypofibrinoly* OR hyperfibrinoly* OR "blood clot lysis" OR "blood clotting" OR "fibrinolytic activity" OR "fibrinolytic system" OR "fibrinolytic shutdown" OR "fibrinolytic resistance" OR "fibrinolytic impairment"

AND

thromboelasto* OR viscoelastic* OR ROTEM OR TEG OR ClotPro OR Quantra OR Sonorheometry

AND

plasminogen or "plasminogen activation" OR "plasminogen activator" OR "tissue plasminogen activator" OR "thrombolytic therapy" OR "fibrinolytic agent" OR alteplase OR urokinase OR anistreplase OR tenecteplase OR streptokinase OR tpa OR "tpa assay"

*Context*

infection OR "systemic inflammatory response syndrome" OR "cytokine release syndrome" OR sepsis OR "septic shock" OR bacteremia OR "bloodstream infection" OR fungemia OR candidemia OR candidiasis OR viremia OR septicemia OR "severe sepsis" OR urosepsis OR "sepsis-associated coagulopathy" OR "severe acute respiratory syndrome" OR "virus pneumonia" OR coronavirus OR covid OR "coronavirus infection" OR "coronavirus disease 2019" OR "COVID-19 pneumonia" OR "covid-19-associated coagulopathy" OR "covid-19 associated coagulopathy"

**Bielefeld Academic Search Engine (BASE)**

*Population*

(N/A)

*Concept*

(fibrino* OR hypofibrinolysis OR "fibrinolytic shutdown" OR "clot lysis")

AND

(thromboelasto* OR viscoelastic* OR ROTEM OR TEG OR ClotPro)

AND

(plasmin* OR "plasminogen activator" OR "tissue plasminogen activator" OR "fibrinolytic agent" OR alteplase OR urokinase)

*Context*

(infection OR "systemic inflammatory response syndrome" OR sepsis OR "septic shock" OR bacteremia OR fungemia OR candidemia OR viremia OR "sepsis-associated coagulopathy" OR "severe acute respiratory syndrome" OR coronavirus OR covid)

**Clinical trial registries**

*Condition*

Infection OR Sepsis OR Septic shock OR Sepsis syndrome OR Systemic Inflammatory Response Syndrome OR COVID-19 Respiratory Infection OR COVID OR COVID19 Infection AND Critical Illness AND Intensive Care Unit

*Intervention*

Viscoelastic OR Thromboelasto* OR ROTEM OR CLOTPRO OR TEG OR Quantra System

*Other*

Fibrinolysis OR Fibrinolysis Shutdown OR Fibrinolytic Deficit OR fibrinolytic defect OR Hyperfibrinolysis OR Hypofibrinolysis OR Fibrinolytic therapy OR coagulation disorder OR Bleeding disorder

**PROSPERO**

*Population*

intensive care or "intensive care medicine" or "intensive care unit*" or ICU or "medical intensive care unit" or "critical ill*" or "critically ill" or "critical care" or "critical care medicine"

*Concept*

fibrinoly* OR fibrino* OR hypofibrinoly* OR hyperfibrinoly* OR "blood clot lysis" OR "blood clotting" OR "fibrinolytic activit*" OR "fibrinolytic system*" OR "fibrinolytic shutdown" OR "fibrinolytic resistance" OR "fibrinolytic impairment"

AND

thromboelasto* OR viscoelastic* OR ROTEM OR TEG OR ClotPro OR Quantra OR Sonorheometry

AND

plasminogen or "plasminogen activation" OR "plasminogen activator" OR "tissue plasminogen activator" OR "thrombolytic therapy" OR "fibrinolytic agent" OR alteplase OR urokinase OR anistreplase OR tenecteplase OR streptokinase OR tpa OR "tpa assay"

*Context*

infection OR "systemic inflammatory response syndrome" OR "cytokine release syndrome" OR sepsis OR "septic shock" OR bacteremia OR "bloodstream infection" OR fungemia OR candidemia OR candidiasis OR viremia OR septicemia OR "severe sepsis" OR urosepsis OR "sepsis-associated coagulopathy" OR "severe acute respiratory syndrome" OR "virus pneumonia" OR coronavirus OR covid OR "coronavirus infection" OR "coronavirus disease 2019" OR "COVID-19 pneumonia" OR "covid-19-associated coagulopathy"

**Table 1: Summary of studies included in scoping review - listed in order of year of publication.** Original fibrinolysis terminology retained as per original reports. Please see Table 2 for definition of fibrinolysis parameters. All assays referred to as fibrinolysis enhanced viscoelastometry (FE-VET).

| First author  Year  Country  [REF] | Study design  Study cohort(s) n= FE-VET Sample size | Demographics  Disease severity  Outcomes | Device  FE-VET agent + dose  ± heparin inhibitor  Fibrinolysis criteria | Assay timing Assay frequency | FE-VET results  Biomarkers | Key findings |
| --- | --- | --- | --- | --- | --- | --- |
| Panigada  2015  Italy  [21] | Prospective observational  **Sepsis*** (43% Septic shock)  n=40  **Healthy controls**  n=40 | **Sepsis**  61 [49–76] yr, male 60%, SOFA 7 [4-10], mortality 20%  *Impaired fibrinolysis:*  SOFA 8 [6-13], overt DIC 13%, mortality 39%  *Normal fibrinolysis:*  SOFA 5 [4-7], overt DIC 0%, mortality 5%  **Healthy controls**  43 [30–55] yr, male 45% | Modified kaolin activated TEG®  Urokinase 80U/ml  + heparin inhibitor  Criterion/a:  Ly30% <64.9% | < 24h of admission  Once | **Sepsis**  TEG Ly30% 0.1 [0–0.9]  UK-TEG Ly30% 70 [29–90]  Elevated PAI-1 and DIC scores, normal TAFI compared to controls  *Impaired fibrinolysis:* 18/40 (45%)  UK-TEG Ly30% 22.9 [0–50.2]  Higher lactate, bilirubin and LDH  PAI-1 did not correlate with UK-TEG  *Normal Fibrinolysis:* 22/40 (55%)  UK-TEG Ly30% 88.5 [76.7–92.4]  **Healthy controls**  TEG Ly30% 1.2 [0.1–2.7]  UK-TEG Ly30% 91.3 [88.5–93.1] | - Increased markers of cellular damage, SOFA scores and mortality in sepsis with impaired fibrinolysis. - Admission UK-TEG Ly30 predicted mortality and higher disease severity. - Sepsis patients hyper-coagulable and hypo-fibrinolytic. - FE-VET revealed a spectrum of fibrinolysis from near normal to severely impaired. |
| Kuiper  2016  Netherlands  [22] | Technical, clinical validation  **Sepsis****  n=20  **Healthy controls**  n=40 | **Sepsis**  N/A  **Healthy controls**  Male 50% | Modified ROTEM delta® EXTEM®  tPA 0, 125 (low) or 175ng/ml (high).  - heparin inhibitor  Criterion/a:  >95% of LOT* and LT* in healthy controls at low and high dose tPA | < 24h of admission  Once | Sepsis  Prolonged LOT and LT at both low and high tPA.  Prolonged CT, higher MCF.  Elevated PAI-1, D-dimer, low tPA and plasminogen activity, normal A2AP & TAFI levels  Healthy controls  LT 35-77 min (low)  LT 25-57 min (high)  LOT 26-49 min (low) & 18-40 min (high) | - FE-VET needed to distinguish fibrinolytic capacity between sepsis and healthy controls. - tPA 175ng/ml had shorter median LT cf. tPA 125ng/ml. - Only sepsis patients with a LT below the median were measurable at 120 min run time using 175 ng/ml tPA. - Sepsis patients showed a hypercoagulable and hypofibrinolytic profile - No hyperfibrinolysis |

| Bakchoul  2020  Germany  [29] | Case report  COVID-19 ICU  n=2 | Case #1:  79 yr, male, pulmonary embolism, ventilatory support, dialysis, SOFA 14, died in ICU  Case #2:  58 yr, male, ventilatory support, ECMO, SOFA 6, survived ICU | ClotPro® tPA-test® tPa 650ng/ml  + heparin inhibitor included  Criterion/a:  N/A | N/A | Case #1:  LT 910 sec  ML 63%  Case #2:  LT 610 sec  ML 97% | - Impaired fibrinolysis in COVID-19. |
| --- | --- | --- | --- | --- | --- | --- |
| Nougier  2020  France  [30] | Prospective observational case control  **COVID-19 ICU**  n=19  **COVID-19 Ward**  n=4  **Healthy controls**  n=10 | **COVID-19 ICU**  63 ± 13 yr, SOFA 5.4 ± 3.1, SAPS II 37.9 ± 13, ventilatory support 67%, dialysis 15%, VTE 29%  **COVID-19 ward**  60 ± 15 yr  **Healthy controls:**  32 ± 14 yr, male 47% | Modified ROTEM delta® EXTEM®  tPA 625 ng/ml tPA  + heparin inhibitor  Criterion/a:  Group comparisons vs. controls | < 3 days of admission  Once | **COVID-19 ICU**  LY30%: 63 ± 39  *VTE positive ICU:*  LY30%: 82 ± 26  *VTE negative ICU:*  LY30%: 37 ± 35  **COVID-19 Ward**  LY30%: 18 ± 35  **Healthy** **Controls**  LY30%: 1.8 ± 3.2 | - Greater fibrinolytic impairment in COVID-19 cf. COVID ward and healthy controls. - Greater impairment of fibrinolysis in patients with VTE. - COVID-19 patients hypercoagulable. - Increased thrombin generation, D-dimer, tPA, PAI-1 and TAFI levels in COVID-19 ICU cf. ward patients. |
| Weiss  2020  France  [31] | Prospective observational  **COVID-19 ICU**  n=5  **Healthy controls**  n=5 | **COVID ICU**  57 ± 15 yr, male 100%, SOFA 9 ± 2, VTE 60%  **Healthy controls**  N/A | Modified ROTEM delta® EXTEM®  tPA 80 ng/mL  + heparin inhibitor  Criterion/a:  Group comparisons vs. controls | Not specified | **COVID ICU**  ML(%) 12 ± 6  Increased MCF in FIBTEM & EXTEM  Elevated fibrinogen levels  **Healthy controls**  ML(%) 94 ± 6.3 | - FE-VET showed normal response in healthy controls and fibrinolysis resistance in COVID-19 ICU. - COVID-19 ICU hyper-coagulable and hypo-fibrinolytic. |
| Zatroch  2020  Hungary  [32] | Case series  **COVID-19 ICU**  n=3 | Patient 1:  62 yr, male, VTE  Patient 2:  84 yr, female, low dose vasopressors, ventilatory support  Patient 3:  80 yr male, high dose vasopressors, dialysis, ventilatory support, died | ClotPro® TPA-test® tPA 650ng/ml  + heparin inhibitor  Criterion/a: Shutdown defined as absent lysis at end of runtime | Variable  Sequential testing in 2 pats | **Patient 1:**  Day 10 LT 315 sec  **Patient 2:**  Day 1: LT 398 sec (intubated)  Day 5: Fibrinolysis shutdown  Day 6: LT 768 sec (improved and extubated)  **Patient 3:**  Day 15: LT 500 sec  Day 19: LT 273 sec | - LT showed more prominent changes than other VET parameters when sequential testing was performed. - Dynamic changes in fibrinolysis detectable using FE-VET. - Hypercoaguable profile in all 3 patients. |
| Bachler  2021  Austria  [33] | Retrospective observational  **COVID-19 ICU**  n=20  **Healthy controls**  n=60 | **COVID-19 ICU**  62 (56-68) yr, male 70%, SOFA 6.5 [3-8.25], ICU LOS 26.5 ± 10 days, mortality 4/20 (20%)  *Impaired fibrinolysis:*  SOFA 7 [3.5-8.75], VTE 1/14 (7%), mortality 4/14 (29%)  *Normal fibrinolysis:*  SOFA 3.5 [3-6.25], VTE 1/6, mortality 0/6  **Healthy controls**  Age 38 (28-46) | ClotPro® tPA-test® tPa 650ng/ml  + heparin inhibitor  Criterion/a:  LT >393s | 8.5 [4.5-15] days of admission  Once | **COVID ICU**  LT 508 sec [365-827]  **Healthy controls**  LT 210 sec [186-261] | - COVID patients displayed hypercoagulable (higher MCF) and hypofibrinolytic profile (longer LT) cf healthy controls. - Impaired fibrinolysis associated with elevated CRP, platelet count, fibrinogen and increased MCF. |
| Duque  2021  Spain  [34] | Case series  **COVID-19 ICU**  n=7  **COVID-19 ward**  n=7  **Healthy controls**  n=15 | **COVID-19 ICU**  57 ± 13 yr, male 29%, SOFA 5 [3], bleeding 57%  VTE 43%, hospital LOS 68±28 days, mortality 29%  **COVID-19 ward**  64 ± 14 yr, male 43% , SOFA 1 [0.5], bleeding 0%, VTE 0%, hospital LOS 10±6 days, mortality 0%  **Healthy controls**  36 ±13.6 yr, male 60% | ClotPro® tPA-test® tPa 650ng/ml  + heparin inhibitor  Criterion/a:  N/A | N/A  Once | **COVID-19 ICU**  LT 400 [93] sec  **COVID-19 ward**  LT 369 [125] sec  **Healthy controls**  LT 201 [63] sec | - Prolonged LT in COVID-19 vs healthy controls. - No difference in LT between ICU and ward patients. - The most hypercoagulable, hypofibrinolytic profile observed in one patient with fatal multiorgan failure. |
| Hammer  2021  Germany  [35] | Retrospective, observational  **COVID-19 ICU**  n=20  **COVID-19 ward**  n=9  **Healthy controls**  n=11 | **COVID-19 ICU**  60 [55-75] yr, male 90%, SOFA 7 [6-11], ECMO 40%, VTE 30%, mortality 25%  **COVID-19 Ward**  44 [14-76] yr, male 44%, mortality 22%  **Healthy controls**  38 [25-47] yr, male 55% | ClotPro® tPA-test® tPA 650ng/ml  + heparin inhibitor  Criterion/a:  Pathologically prolonged LT >2x SD above median LT in healthy controls | N/A  Once | **COVID-19 ICU**  LT 299 [238-475]  ML% 96 [86 - 97]  **COVID-19 Ward**  LT 310 [291-436]  ML% 97 [96-98]  **Healthy controls**  LT 177 [169-195]  ML% 94.5 [94.95]  COVID-19 ICU: higher PAI-1, tPA, similar plasminogen activity and reduced A2AP vs. controls  Positive correlation between PAI-1 activity and LT (r = 0.70) | - Prolonged LT in COVID-19 vs controls, but not between COVID-19 patients. - LT most prominent to differentiate COVID-19 ICU and healthy controls. - No significant differences observed in FE-VET parameters based on VTE or mortality. - Hyperfibrinolysis not observed. |
| Heinz  2021  Germany  [36] | Prospective observational  **COVID-19 ICU**  n=27  **Healthy controls**  n=12 | **COVID-19 ICU**  60 ± 13 yr, male 78% , ventilatory support 100%, dialysis 52%, SAPS II 42 ± 10  **Healthy controls**  38 ± 6 yr, male 50% | ClotPro® tPA-test® tPA 650ng/ml  + heparin inhibitor  Criterion/a:  Normal LT 145 – 438 sec | 7 ± 3.5 days  after ICU admission  Once | **COVID-19 ICU**  LT 530s ± 327  Elevated CRP, PCT, IL6, wVF and factor VIII, IX. Decreased protein S  **Healthy controls**  LT 211 ± 80 sec | - Prolonged LT and CT in COVID-19 ICU, increased MCF in EX, FIB, TPA tests vs. controls. - Platelet aggregability not increased (Multiplate). |
| Hulshof  2021  Netherlands  [38] | Prospective longitudinal cohort  **COVID-19 ICU**  n=36 | **COVID ICU**  61 [55–70] yr, male 81%, APACHE II 15 [13–18], SAPS II 40 [29-44], SOFA 7 [6.5-7], ventilatory sup-port 100%, dialysis 28%, ECMO 17%, ICU LOS 34 [21-42] days ,VTE 56%, mortality 19% | Modified ROTEM delta® EXTEM®  tPA 125ng/ml  - heparin inhibitor  Criterion/a:  N/A | 18 [6-25] days after intubation  Serial samples twice weekly | **COVID ICU**  Median LOT >49 min over 6 weeks  Median LT 120 min over 6 weeks  LT >120 min runtime in 56% of assays | - Longitudinal analysis (6 wk) demonstrated persisting hypercoagulability (MCF, FIBTEM, EXTEM) and hypofibrinolysis despite anticoagulant treatment. |
| Hulshof  2021  Netherlands  [37] | Prospective longitudinal cohort  **COVID-19 ICU**  n=22 (11 patients lost to follow up) | **COVID-19 ICU**  61 [57-69] yr, male 77%, APACHE II 15.5 [13-18], SAPS II 37.5 [29-43], ven-tilatory support 100%, dialysis 9%, ECMO 14%, ICU LOS 34 [22-42] days, VTE in ICU 64% | Modified ROTEM delta® EXTEM®  tPA 125ng/ml  - heparin inhibitor  Criterion/a:  N/A | 25 [18-40] days after ICU ad-mission, -1 [-4 - 0] days after ICU discharge, 188 [171-192] days after ICU dis-charge. | **ICU Discharge**  LT 7200 [6144-7200] sec  LOT 3690s [2963-4418] sec  **6 month follow up**  LT 3138 [2591-4389] sec  LOT 1786 [1465-2650] sec | - Hypercoagulable or hypofibrinolytic profile normalised 6 months after ICU discharge. - No difference in FE-VET parameters at follow up for patients with and without VTE. |
| Maier  2021  USA  [39] | Prospective observational  **COVID-19 ICU**  n=8    **COVID-19 Ward**  n=6  **Healthy controls**  n=14 | **COVID-19**  N/A  **Healthy controls**  Male 50% | kaolin activated TEG 5000®  platelet poor plasma +/- tPA 4 nM (approx. 300 ng/mL)  + heparin inhibitor  Criterion/a:  N/A | 8 (1-8) days after admission | **COVID-19**  CLT 48.8±16.3 min  Ly30% 37.9±16.5 min  Elevated MA, elevated fibrinogen  **Healthy**  CLT 30.5 ± 15.4 min  Ly30% 58.9±18.3 | - Significant difference in Ly30% & CLT between groups - Fibrinolytic impairment only evident when tPA added to assay. - Prolonged fibrinolysis times observed in COVID-19 patients cf. controls. |
| Panigada  2021  Italy  [40] | Prospective observational  **COVID-19 ICU**  n=22  **Healthy controls**  n=10 | **COVID-19 ICU**  57 [46-65] yr, SOFA 3 [3-4]  **Healthy controls**  43 [30 – 54 yr] | Modified kaolin activated TEG®  +/- urokinase 80U/ml  + heparin inhibitor  Criterion/a: Hypofibrinolysis Ly30 <64.9% with urokinase | N/A  Once | **COVID-19 ICU**  Standard TEG  Ly30% 0.1 [0.0-1.9]  (64% hypofibrinolysis)  UK-TEG  Ly30% 87.7 [80.5-90.2]  (14% hypofibrinolysis)  **Healthy controls**  TEG Ly30% 1.2 [0.1-2.7]  UK-TEG Ly30% 91.3 [88.5-93.1] | - Hypofibrinolysis common in COVID-19 patients with standard TEG (64%) and infrequent with urokinase TEG assay (14%). - Urokinase increased sensitivity to identify true hypofibrinolysis. |
| Schrick  2021  Hungary  [41] | Prospective observational  **COVID-19 ICU**  n=21  **Age matched controls**  n=21 | **COVID-19 ICU**  69 [52-71] yr, male 57% , ICU mortality 86%  **Controls**  67 [63–69] yr, male 52% | ClotPro® TPA-test® tPA 650ng/ml  + heparin inhibitor  Criterion/a:  Decreased fibrinolytic response at LT >393s | N/A  Once | **COVID-19 ICU**  Patients grouped by aspirin responsiveness (Multiplate) and fibrinolytic response.  8/21 aspirin non-responders with  decreased fibrinolytic response in 9/21  TPA LT prolonged in aspirin non-responders compared to aspirin responders  Increased D-dimer and vWF, lower plasminogen levels cf. controls | - Stronger clots in aspirin non-responders cf. responders. - Decreased fibrinolytic response in aspirin non-responders cf. responders. - No association between platelet reactivity and VTE status (38% VTE in COVID-19). |
| Heubner  2022  Germany  [42] | Retrospective observational  **COVID-19 ICU**  n=55 | **COVID-19 ICU**  65 [58-69] yr, male 78% , ventilatory support 100%, dialysis 5.5%, ECMO 40%, VTE 51, hos-pital mortality 55%  *Survivors 25/55:*  63 [57-68] yr, male 68% , SOFA 10 [9-11], bleeding 20%, VTE 56%  *Non-survivors 30/55:*  68 [61-71] yr, male 87% , SOFA 12 [11-14], blee-ding 17%, VTE 43% | ClotPro® tPA-test® tPA 650ng/ml  + heparin inhibitor  Criterion/a:  Fibrinolysis shutdown if ML<50% at 40 min | 17 [3-38] days after symptoms onset  Once | *Survivors:*  LT 453 [283-560] sec  *Non-survivors:*  LT 432 [345-538] sec  *VTE positive:*  LT 460 [350-560] sec  *VTE negative:*  LT 359 [287-521] sec | - FE-VET did not differentiate survivors vs. non-survivors - Impaired fibrinolysis in VTE patients - Hypercoagulable and hypofibrinolytic state associated ARDS disease severity - FE-VET did not predict bleeding complications |
| Nagy  2022  Netherlands  [43] | Poster Abstract:  Nested case control  **COVID-19 ICU**  No intervention, *CTRL:*  n=12  Dexamethasone *DEXA:*  n=12  Dexamethasone + tocilizumab, *DEXA+TOCI*  n=12 | *CTRL:*  62 [59-68], male 83%, APACHE II 16 [13-20.75], ICU LOS: 22 [19-34] days, mortality 42%  *DEXA:*  60 [57-65], male 83%, APACHE II: 15.5 [11.75-18.5], ICU LOS: 25 [20-37] days, mortality 58%  *DEXA+TOCI:*  60 [59-63] yr, male 83%, APACHE II: 15 [12.5-17.5]  ICU LOS: 43 [34-60] days, mortality 33% | Modified ROTEM delta® EXTEM®  tPA 125ng/ml  - heparin inhibitor  Criterion/a:  N/A | 3 weeks after treatment  Weekly | *Week 1:*  CTRL: LT 7200 [6637-7200] sec  DEXA: LT 7200 [6268-7200] sec  DEXA+TOCI: LT 5183 [4558-5888] sec  *Week 2:*  No difference between groups  *Week 3:*  LT in CTRL decreased  LT in DEXA+TOCI increased  PAI-1 levels increased in DEXA+TOCI but decreased in CTRL and DEXA over 3 weeks. | - Shortest LT and lowest PAI-1 levels at week 1 in DEXA + TOCI suggesting improved fibrinolysis. - Monitoring fibrinolysis may reflect therapeutic effects. |
| Coupland  2023  Australia  [23] | Prospective observational + exploratory interventional  **COVID-19 ICU**  n=69  **Non COVID-19 ICU**  n=36  **Healthy controls**  n=20 | **COVID-19 ICU**  65 [52-71] yr, male 62% , APACHE III: 66 [49-78], ventilatory support 45%, ICU LOS 6.7 [2.8-12.9] days, hospital mortality 28%  **Non COVID-19 ICU**  59 [40-71] yr, male 69%, sepsis 28%, trauma 22%, APACHE III: 67 [53-81], ventilatory support 61%, ICU LOS 6.4 [2.2-19] days, hospital mortality 19% | ClotPro® TPA-test® tPA 650 ng/ml  + heparin inhibitor  Criterion/a:  Fibrinolysis resistance if LT > 300 sec (>4 SD above mean of healthy controls),  Fibrinolysis shutdown if absent clot lysis during 2400 sec runtime. | 1 [1-2] days after admission  Once | **COVID-19 ICU**  LT 302s [252–364]  Fibrinolysis resistance 51%  **Non COVID-19 ICU**  LT 338 [270–432] sec  Fibrinolysis resistance 64% | - Fibrinolysis resistance prevalent in critically ill population. - Additional tPA reduced fibrinolysis resistance ex vivo if LT <1000 sec. - Additional plasminogen reduced fibrinolysis resistance if LT>1000 sec. - Fibrinolysis resistance in one case of bacterial pneumonia given low dose alteplase infusion over 24 hr demonstrated gradual reduction (25%) in TPA-test LT with rebound after alteplase ceased. - FE-VET can investigate cause of fibrinolytic impairment, guide therapy and monitor treatment effects. |
| Forgács  2023  Hungary  [44] | Case report  **COVID-19 ICU**  n=1 | 48 yr, male, APACHE II 18, SOFA 9, ventilatory support, prone position, dialysis, therapeutic anticoagulation | ClotPro® tPA-test® tPA 650ng/ml  + heparin inhibitor  Criterion/a:  Fibrinolysis resis-tance if LT > 300 sec  Fibrinolysis shut-down if absent clot lysis during runtime | Daily in ICU  Every 3 hr during systemic throm-bolysis to guide alteplase dosing | Day 1: LT 325 sec  Day 2: LT 755 sec  Day 3: Shutdown  *Developed portal vein thrombosis on Day 3 despite therapeutic anticoagulation.*  Day 4: LT 170 sec after systemic thrombolysis | - Developed algorithm to cal-culate systemic alteplase infusion and fresh frozen plasma transfusions tit-rated to reverse fibrinolytic shutdown. - Thrombolysis associated with improved lung gas ex-change, lactate, liver func-tion and restored portal vein patency. |
| Thaler  2023  Germany  [45] | Prospective observational  **COVID-19 ICU**  n=14  **COVID-19 Ward**  n=46 | **COVID-19 ICU**  61 [45-73] yr, male 71%, ventilatory support 50%, ECMO 8%  **COVID-19 Ward**  60 [44-65] yr, male 72% | ClotPro® TPA-test® tPA 650ng/ml  + heparin inhibitor  Criterion/a:  N/A | At ≤ 8 hr and at ≤ 24 hr after hos-pital admission  Twice | **COVID-19 ICU**  *At hospital admission*  LT 249 [218-280] sec  *At ICU admission*  LT 273 [232-389] sec  **COVID-19 Ward**  *At hospital admission*  LT 266 [216-308] sec  MCF (mm) increased between hospital and ICU admission  Patients needing ICU admission had increased D-dimer and PAI-1 levels | - No difference in any VET variables between COVID-19 ICU or ward patients at hospital admission. - Increase in MCF and LT at ICU admission cf. hospital admission. - FE-VET did not predict ICU level care. |
| Hulshof  2024  Netherlands  [46] | Prospective longitudinal cohort  **COVID-19 ICU**  n=138 | **COVID-19 ICU**  64y ± 9 yr, male 75%, APACHE II 15 ± 5, ven-tilatory support 100%, dialysis 6%, ECMO 4%, ICU LOS 14 [8–22] days, ICU mortality 41% | Modified ROTEM delta® EXTEM®  tPA 125ng/ml  - heparin inhibitor  Criterion/a:  Group comparisons survivors vs. non-survivors | 15 [8-24] days after admission  ROTEM at least once weekly  3 [2-5] ROTEM measurements per patient | LOT, LT, PT and MCF increased over time in non-survivors (41%) cf. survivors  Prolonged CT and increased fibrinogen in non-survivors cf. survivors  EXTEM and FIBTEM CT at intubation associated with increased 45-day ICU mortality. | - Serial VET showed dynamic changes, with worsening hypercoagulability and fibrinolytic impairment in non-survivors. - All significant associations persisted after adjustment for demographics and APACHE score. |
| Rezigue  2024  France  [47] | Prospective experimental  **COVID-19 ICU**  n=15  **Healthy controls**  n=30 | **COVID-19 ICU**  N/A  **Healthy controls**  32 ± 14 yr, male 47% | Modified ROTEM delta® EXTEM®  tPA 625 ng/ml  + heparin inhibitor  Criteria:  Group comparisons vs. healthy controls | N/A | Paired comparison of whole blood samples treated *ex-vivo* with hydroxyethyl starch (HES) or saline from the same patient:  Decreased Ly30% in hydroxyethyl starch treated samples cf. control saline samples. | - HES enhanced tPA-induced fibrinolysis in patients with severe COVID-19 by redu-ced fibrin polymerisation. |
| Brewer  2024  Denmark  [48] | Prospective observational cohort  **Sepsis*****  n=30, 53% septic shock  **Non-sepsis**  n=129  **Healthy controls**  n=38 | **Sepsis**  SOFA 9 [7-11], DIC score 3 [2-4], VTE 20%, blee-ding 70%, mortality 40%  **Non-sepsis**  SOFA 9 [5-11], DIC score 2 [2-3], VTE 5%, bleeding 69%, mortality 19%  *Impaired fibrinolysis:* (61%), 67 [54–74] yr, male 37%, SOFA 10 [7-11], ventilatory support 81%, dialysis 16%, ECMO 9%, VTE 11%, bleeding 77%, ICU LOS 4 [2-8] days, mortality 31%  *Normal fibrinolysis:* (39%), 63 [56–71], male 34% , SOFA 7 [4-10], ven-tilatory support 53%, dialysis 5%, ECMO 2%, VTE 3%, bleeding 57%, ICU LOS 2 [1-5] days, mortality 11% | Modified ROTEM delta® EXTEM® with tPA 125ng/ml tPA  + heparin inhibitor  Criterion/a:  Fibrinolytic impairment LT > 97.5^th^ percentile cf healthy control (3000 sec)  Hyperfibrinolysis LT <as LT 2.5^th^ percentile cf. healthy control | Single analysis performed within 1^st^ 24h of ICU admission | **Sepsis**  LT 3600 [3352-3600] sec  Fibrinolytic impairment 80%  **Non-sepsis**  LT 3374s [2175-3600]  Fibrinolytic impairment 57%  Hyperfibrinolysis (n = 2) | - Impaired fibrinolysis most common in the sepsis group, but also prevalent in non-sepsis patients, cf. healthy controls. - Overall, impaired fibrino-lysis associated with higher SOFA scores, lactate, CRP, bleeding episodes, require-ments for vasopressor, mechanical ventilation renal replacement therapy, and longer ICU length of stay. - decreased30-day survival in patients with impaired fibrinolysis cf. normal fibrinolysis in Kaplan-Meier analysis. |
| Scarlatescu  2024  Romania  [16] | Technical, clinical validation  **Sepsis/septic shock*****  n=30  **Healthy controls**  n=30 | **Sepsis/septic shock**  72 [67.5-80] yr, male 47%, SOFA 9.2 ± 1.9, ICU mortality 70%  **Healthy controls**  50 [42-57] yr, male 37% | Modified ROTEM delta EXTEM  tPA at varying concentrations (0, 100, 175, or 300 ng/mL)  + heparin inhibitor  Criterion/a:  CLI60 reference range in healthy controls 57-97%.  Fibrinolysis resistance CLI60 ≥98% in EXTEM-tPA 175ng/ml | Within 24-36h after sepsis diagnosis | **Sepsis/septic shock**  Impaired fibrinolysis 37%  EX-tPA 175 CLI60%: 96 [93-98]  EX t-AUCi 1845 [1680-2107] sec  **Healthy controls**  EX-tPA 175 CLI60% 89 [83-92]  EX t-AUCi 1449 [1353-1601]  t-AUCi obtained from standard EXTEM (non FE-VET) of >1962 sec had 81.8% sensitivity and 83.7% specificity for RF prediction  Sepsis patients had higher PAI-1, plasmin- α2-anti-plasmin complex, fibrinogen levels, lower plasmino-gen levels and similar TAFI levels. | - Impaired fibrinolysis in sepsis cf. healthy controls. - In sepsis patients, only tPA 300 ng/ml caused a significant reduction in lysis parameters compared to lower tPA concentrations and revealed a wider range of fibrinolytic function. - Higher tPA concentrations needed to determine the spectrum of fibrinolysis resistance, with complete clot lysis and shortened runtime. |

**Septic shock 2005, **Surviving sepsis guideline 2012, ***Sepsis-3 definition*

Abbreviations: APACHE = Acute Physiology and Chronic Health Evaluation; SOFA = Sequential Organ Failure Assessment; VTE = Venous ThromboEmbolism; ECMO = ExtraCorporeal Membrane Oxygenation; LOS = Length Of Stay
